# Metabolomic Biomarkers Predict Long-term Physical Function in Survivors of Acute Respiratory Failure

**DOI:** 10.21203/rs.3.rs-7394034/v1

**Published:** 2025-11-02

**Authors:** Adeyeye I. Haastrup, Justin T. Roberts, Sheetal Gandotra, Emily M. Hartsell, Grant T. Daly, Viktor M. Pastukh, Lina D. Purcell, Ryan G. Benton, D. Clark Files, Troy Stevens, Mark N. Gillespie, Peter E. Morris, Raymond J. Langley

**Affiliations:** University of South Alabama Frederick P. Whiddon College of Medicine; University of South Alabama Frederick P. Whiddon College of Medicine; University of Alabama-Birmingham College of Medicine; University of South Alabama Frederick P. Whiddon College of Medicine; University of South Alabama Frederick P. Whiddon College of Medicine; University of South Alabama Frederick P. Whiddon College of Medicine; Wake Forest Baptist Medical Center; University of South Alabama School of Computing; Wake Forest Baptist Medical Center; University of South Alabama Frederick P. Whiddon College of Medicine; University of South Alabama Frederick P. Whiddon College of Medicine; University of Alabama-Birmingham College of Medicine; University of South Alabama Frederick P. Whiddon College of Medicine

**Keywords:** Acute respiratory failure, post intensive care syndrome, physical function, metabolite feature selection for patient classification, logistic regression

## Abstract

**Introduction:**

Acute respiratory failure (ARF) often leads to post-intensive care syndrome, including persistent physical impairments after ICU discharge. Emerging evidence suggests that mitochondrial bioenergetic dysfunction, detectable through metabolomic profiling, may contribute to poor recovery.

**Methods:**

We performed a retrospective study comprising of untargeted metabolomic profiling using ultrahigh performance liquid chromatography–mass spectrometry (UHPLC-MS) on serial serum samples from 70 ARF patients taken at ICU admission, during hospitalization and at discharge. Physical function was assessed post-discharge using the Short Physical Performance Battery (SPPB). Correlation and logistic regression analyses were performed to identify metabolomic predictors of six-month physical function outcomes.

**Results:**

Patients with poor SPPB scores exhibited dysregulation in bioenergetic metabolite levels, as well as fatty acid oxidation, glycerophospholipid metabolism, bile acid biosynthesis and amino acid metabolism. These metabolic changes were not explained by initial disease severity (APACHE III scores) or comorbidities. In contrast, several metabolites measured at discharge were predictive of SPPB scores with an AUROC of 0.88 after cross validation.

**Conclusion:**

Our findings highlight persistent metabolic dysfunction at discharge, particularly in pathways related to bioenergetics. To our knowledge, this is the first study to employ a metabolite-based machine learning model to predict ARF survivors physical function outcomes using serum metabolites measured at discharge. Further insights on dysregulated pathways suggest that nutritional interventions targeting these metabolic pathways, such as supplementation with β-alanine, could potentially improve post-ICU recovery outcomes.

## Introduction

Acute respiratory failure (ARF) is a life-threatening condition characterized by inadequate blood oxygenation with or without hypercapnia [1,2]. It is often associated with critical conditions such as sepsis, chronic obstructive pulmonary disease (COPD), and acute respiratory distress syndrome (ARDS), and imposes a substantial socioeconomic burden on affected patients and society as a whole [3,4]. A multinational study revealed that more than 50% of critically ill patients in the ICU have or will develop ARF, which places them at a 40% risk of mortality [5].

Survivors of ARF often face multiple physical and cognitive difficulties that are collectively termed post-intensive care syndrome (PICS) after ICU discharge [6–9]. About 46% of ARF survivors are at high risk of readmission or dying within 3–4 months following initial ICU discharge which may be due to PICS-related disabilities [10,11]. Persistent physical disabilities after discharge from the ICU [6,12,13], reflecting impairments in muscle strength, mobility, and overall function long after the acute illness [14], are core components of PICs. In survivors of COVID-19-related ARDS, the incidence of physical function impairment is approximately 74% at 1 year after ICU treatment [15]. The quality of life decrement for these patients is influenced significantly by various factors, including underlying health conditions, demographics, ICU treatment, and the medical, pharmacological, and rehabilitative follow-up treatments [16–19].

The Short Physical Performance Battery (SPPB) is a tool commonly used to measure physical performance in the elderly [20]. It has been shown to correlate with various important health outcomes, including the ability to perform daily activities, risk of hospital admission or readmission, overall functional status, and mortality rates in critically ill and elderly subjects [21,22]. While the etiology of ARF is reasonably well understood, pathophysiologic mechanisms contributing to persistent poor physical performance in post-ICU patients, as well as reliable tools to predict long-term functional outcomes remain elusive [12,23]. The long-term quality of life outcomes for ARF survivors, identified as a key research priority by critical care professional societies and pulmonary physicians, emphasizes the urgent need for research focused on delineating the pathophysiologic changes that occur at the molecular level [24,25].

Metabolomics is increasingly utilized to profile interactions at a system level in patients with critical illness [26,27]. This approach may enable a comprehensive and holistic understanding of the disease process as well as improvements in diagnosis, assessing response to therapy, monitoring disease progression, pathological mechanisms, biomarker, and pharmacologic target discovery [28].

Our previous studies identified bioenergetic dysregulation as a key factor in non-survivors of sepsis, ARF, and acute kidney injury (AKI) [29–34]. The prominently affected pathways in non-survivors were bioenergetic, specifically NAD^+^ metabolism and β-oxidation, which are essential for mitochondrial bioenergetics, and these were accompanied by disruptions in acetylcarnitine, glutathione, bile acid, steroid, and fatty acid metabolism[30]. Building on this, because physical exercise performance is inherently linked to mitochondrial bioenergetics [35,36], we postulated that the dysregulation in bioenergetic pathways may follow a continuum, with varying degrees of severity correlating with a range of outcomes. For example, those with the most pronounced metabolic disruptions did not survive, while survivors with poor outcomes experience milder but still impactful bioenergetic dysregulation, that potentially impairs physical performance. In this study, we hypothesized that poor physical function in ARF survivors is associated with, and predicted by, bioenergetic dysfunction, as reflected by metabolomic and mitochondrial biomarkers.

## Methods

This retrospective study involved ARF patients enrolled in the Standardized Rehabilitation for ICU Patients with Acute Respiratory Failure clinical trial at Wake Forest Baptist Medical Center in North Carolina (ClinicalTrials.gov Identifier, NCT00976833; registration date, 2009–09-11). Institutional review board approval was obtained, and informed consent was provided by patients or their legal representatives. ICU patients were enrolled based on a clinical diagnosis of ARF made by the attending physicians, following the inclusion and exclusion criteria previously outlined [30,37]. All patients were treated according to standard of care practices, comprising a variety of pharmacological therapies. To ensure comparability between the groups, the patients were matched for age, race, and sex. Demographics of the selected patients are summarized in Table 1. Global metabolomics was performed on patients’ serum samples at three time points: ICU admission (n = 70), five days post-admission (n = 40), and ICU discharge (n = 69). Before blood collection from a central line, the catheter was flushed with sterile saline and a waste sample was drawn to remove residual fluids or anticoagulants, ensuring uncontaminated samples. Samples were collected at enrollment, day 5 post-enrollment, and at ICU discharge. The smaller number of samples at day 5 and at discharge reflects both study design and patient availability. We anticipated that this timepoint would provide limited clinical utility beyond showing temporal trends, so we prioritized enrollment and discharge samples to maximize predictive power. Furthermore, several patients in both good and poor outcomes were discharged prior to day 5. There was one sample at discharge that was not measured by Metabolon and therefore removed from the analysis. At discharge, and at two-, four-, and six-months following ICU admission, we assessed physical function using the SPPB, an objective measure that evaluates gait speed, balance, and lower extremity strength [38].

Serum Metabolomics: Extraction and quantification of serum metabolites were performed by Metabolon Inc [30]. Briefly, 100ul of each sample was prepared with recovery standards using the automated MicroLab STAR^®^ system (Hamilton Company). Sample runs were performed on C18 and HILIC columns coupled to a Thermo Scientific Q-Exactive high-resolution/accurate mass spectrometer with the orbitrap mass analyzer operated at 35,000 mass resolution in negative and positive ionization modes. Metabolon’s hardware and proprietary software were used for spectral data extraction and compound identification. Artifacts and background noise were removed. Peaks were quantified using the area-under-the-curve method. Specifically, identifications are based on a narrow retention index, mass accuracy within ± 10 ppm, and MS/MS spectral matching to authentic standards (forward and reverse scores). The scaled intensity of metabolites was used to provide semi-quantitative metabolite values, wherein each value quantifies the relative concentration of a metabolite in a sample. The mass area was normalized to account for sample volume, blanks, quality controls, and internal standards. Variance filtering for repeatability (using QC samples) was performed using internal standards and endogenous metabolites, with relative standard deviation thresholds of 3% and 9%, respectively.

Metabolomic Data Processing and Statistical Comparisons: Metabolites with greater than 30% missing points were excluded from further processing [39,40]. Missing data points were imputed with mechanism-aware imputation algorithm [40]. A Shapiro-Wilk test performed on the data before and after imputation gave p-value < 0.05, indicating non-normal distribution of the metabolite data. To improve signal-to-noise ratio and enhance downstream modeling, metabolites with a relative median absolute deviation less than 0.25, representing near-constant features with limited statistical power, were excluded [41,42]. Metabolite intensities were transformed to log scale in order reduce skewness. To explore differences in metabolite levels between functional outcome groups, we applied the Wilcoxon rank sum test (two-sided) to compare survivors with poor (SPPB ≤ 6) and good (SPPB ≥ 7) scores. Metabolites with p < 0.05 were considered to show the strongest differences and are hereafter referred to as “differential metabolites”. We explored false discovery rate (FDR) correction using multiple methods including the Benjamini-Yekutieli procedure; however, due to the highly discrete distribution of p-values, modest sample size and limited variability among test statistics, the adjustment yielded uniformly non-significant results. As such, FDR-adjusted results were not used for inference. Metabolomic profiles were analyzed separately at three timepoints: day 1, day 5 post admission, and at hospital discharge. Multivariate hierarchical clustering of differential metabolites was performed to assess temporal changes in metabolomic profiles between good and poor physical function groups across the three time points. The data were analyzed using custom-developed scripts in RStudio version 4.4.1 [43].

Statistical analysis of the demographic data was conducted using Wilcoxon rank-sum test for continuous variables and Fisher’s exact test for categorical variables. A p-value < 0.05 was considered significant when comparing poor and good physical performance groups.

Chemical Similarity Enrichment, Pathway and Meta-Analysis: Chemical similarity enrichment analysis was performed using ChemRICH, which enabled us to identify classes of compounds that were up- or downregulated. ChemRICH also offers the advantage of identifying clusters of chemically similar metabolites, revealing potential new metabolite sets that may be relevant [44]. Pathway analysis was performed using MetaboAnalyst 6.0 focusing on differential metabolites to identify specific dysregulated pathways in subjects with poor physical function [45,46]. A meta-analysis was conducted to evaluate the association of self-declared race (white or black) and gender (male or female) with differential metabolites that were either upregulated or downregulated. In addition, Fisher’s Exact Test was used to assess the association between physical function outcomes, categorized as good vs. poor SPPB, and clinical parameters including APACHE III score, body mass index (BMI), mean arterial pressure (MAP), heart rate (pulse), respiratory rate, fraction of inspired oxygen (FiO_2_), and arterial partial pressure of carbon dioxide (PaCO_2_), as well as comorbidities present at admission.

Metabolite Feature Selection, Classification, and Model Validation: As indicated subsequently, the highest number of differential metabolites and dysregulated pathways was observed in the poor SPPB group at discharge. To streamline relevant features for sample classification, we applied a filtering process on differential metabolites measured at discharge. Log transformed data were mean-centered and scaled to unit variance. Using the publicly available online version of MetaboAnalyst^™^ software, a Partial Least Squares Discriminant Analysis (PLS-DA) was performed to classify the patients into the respective SPPB categories, the classification being executed utilizing the biomarker candidates comprising only the differential metabolites. Additionally, a supervised machine learning, Random Forest classification model was implemented to rank biomarker candidates features based on their discriminatory power, using the randomForest and caret R packages [47,48]. Ten metabolites identified at this stage were subjected to Spearman correlation analysis, to eliminate redundant features exhibiting high collinearity or duplicative discriminatory effects. A correlation threshold of 0.8 was defined, and biomarker pairs exhibiting absolute correlation values exceeding this threshold were identified as highly correlated features. Furthermore, a Bayesian logistic regression model incorporating leave-one-out cross-validation was utilized (rstanarm and loo packages in R) to assess the impact of inclusion or exclusion of metabolites on model performance [49,50]. Based on this iterative evaluation, seven metabolite biomarkers identified as the most predictive of patients’ physical function were selected for standard logistic regression analysis with 5-fold cross-validation. Given the strong performance of the standard model, we further evaluated the predictive power of these metabolites using a Bayesian logistic regression framework for comparison. To evaluate the generalizability of the model to unseen data, a 5-fold cross-validation procedure was conducted. To validate the robustness and stability of the model across variable data partitions, a repeated k-fold cross-validation procedure was implemented, through twenty repetitions of 5-fold cross-validation. The execution parameters included five independent Markov chains, each with 2,000 iterations and a fixed random seed of 12345 for reproducibility.

## Results

To determine if bioenergetic metabolites are predictive of six-month physical function in survivors of ARF, we performed broad spectrum metabolomic analysis of serum samples collected at enrollment (day 1), day 5 post enrollment, and patient discharge. Seventy patients were selected from the Standardized Rehabilitation for ICU Patients with Acute Respiratory Failure cohort. Patients were matched for age, race, and sex (Table 1). Half the patients were determined to have a “good” physical performance, (SPPB ≥ 7), while the other half had a “poor” physical performance (SPPB ≤ 6). Demographics of the selected patients are summarized in Table 1.

Mass spectrometry analysis was performed by Metabolon Inc. as described in the methods. In total, 758 named biochemicals and 151 unnamed biochemicals were detected (total: 909 biochemicals). After removing near-constant features and restricting to named compounds, 742 biochemicals were retained for analysis. Samples identified as outliers by Metabolon Inc. were removed from the statistical analysis, reducing the number of samples to 66, 39, and 64 on day 1, day 5, and at discharge, respectively. Further analysis was performed on named biochemicals.

**Figure 1:**
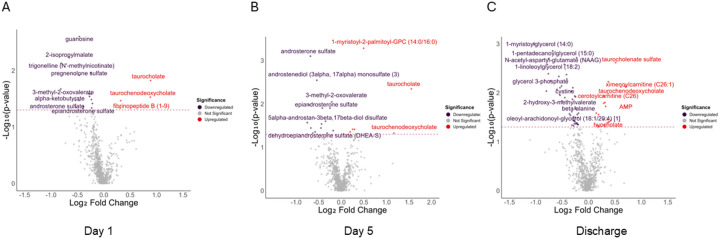
Volcano Plots Depicting Differential Serum Metabolites at Three Clinical Time Points. Volcano plots of metabolomic data comparing differential abundance of metabolites (p-value < 0.05) at three clinical time points among ARF survivors: (A) day 1 (ICU admission), 21 differential metabolites; (B) day 5 (post-admission), 21 differential metabolites; (C) at hospital discharge, 67 differential metabolites. Red points indicate upregulated differential metabolites, and purple points indicate those downregulated in participants with poor SPPB scores compared to those with good SPPB scores. Statistical threshold is marked by a dashed horizontal line (Wilcoxon rank sum p<0.05).

Univariate and Multivariate Analysis Results: On day one, 21 serum metabolites concentrations were identified as having the strongest difference (p < 0.05) between outcome groups. Patients with poorer physical outcomes exhibited a marked reduction in bioenergetic-related metabolites, including trigonelline, a metabolite of NAD^+^, which plays a key role in cellular energy metabolism (Fig. 1A) [51].

At day 5 post-admission, 21 serum metabolites differentiated patients with poor and good physical performance. Androgens play crucial roles in bioenergetics [52,53]. Amongst the differential metabolites, five androgen-related metabolites were lower in patients who had poor physical function (dehydroepiandrosterone sulfate [DHEA-S], androsterone sulfate, epiandrosterone sulfate, 5-alpha-androstan-3beta,17beta-diol disulfate, and androstenediol [3alpha, 17alpha] monosulfate) (Fig. 1B).

Remarkably, 67 differential metabolites distinguished patients with poor versus good physical function at discharge. Among ARF survivors with good SPPB scores, 22 differential metabolites were decreased at discharge, compared to only 6 on day 5 and 4 on day 1. In contrast, survivors with poor SPPB scores showed 45 differential metabolite with decreased concentrations at discharge, 15 at day 5, and 17 at day 1. This highlights a noticeable shift in the metabolomic profile by the time of hospital discharge.

Bioenergetic metabolites such as hydroxyl fatty acids, fructose, glycerides, alpha-ketoglutarate, and glycerol-3-phosphate were reduced in patients with poor physical function (Fig. 1C).

Although the primary aim of this study was to identify predictive metabolite biomarkers and develop an FDR-independent predictive model, initial analyses revealed several metabolites with nominal significance (p < 0.05); however, these did not pass the Benjamini-Yakutieli correction (adjusted p > 0.05).

Multivariate analysis of the metabolite trends between the good and poor SPPB score groups across time point showed some temporal differences reflecting dynamic metabolic shifts across the three time points (Additional file1: figure S1).

Chemical Similarity Enrichment and Pathway Analysis Results: We performed chemical and pathway enrichment analyses to evaluate chemical similarities among enriched metabolites, identify trends in metabolic pathway alterations during hospitalization, and highlight potential pathways that may be targeted for therapeutic intervention.

**Figure 2:**
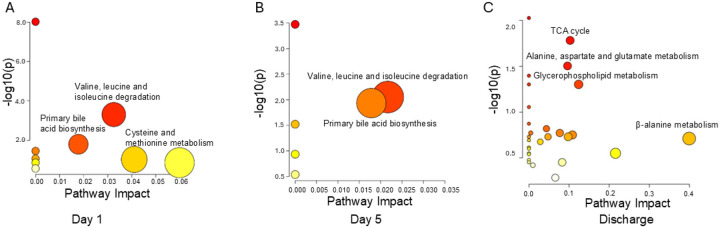
Enriched Metabolic Pathways in ARF Survivors Across Three Clinical Time Points from ICU Admission to Discharge. Pathway enrichment based on differential metabolites measured at three clinical time points: (A) ICU admission (day 1), (B) day 5 post-admission, and (C) hospital discharge. Circle size corresponds to the relative impact of each metabolic pathway, and circle color intensity (yellow to red) reflects statistical significance (−log10(p-value)).

Analysis of the metabolomic panel on day 1 reveals disruption in the catabolism of valine, leucine, and isoleucine in ARF survivors with poor SPPB scores (Fig. 2A). These branched-chain amino acids are essential for muscle metabolism and mitochondrial energy production [54]. Pathway analysis further underscores slight perturbations in cysteine and methionine metabolism. ChemRICH analysis highlights the decreased keto acids (branched-chain amino acid metabolites) and increased cholic acids (bile acids) which corresponds with metabolic pathways identified as impacted by the pathway analysis with MetaboAnalyst (Additional file 1: Figure S2 A).

On day five, pathway analysis demonstrates dysregulation in both primary bile acid biosynthesis and amino acid metabolism in group of ARF survivors with poor SPPB scores (Fig. 2B). Similar to day one, ChemRICH analysis only revealed decreased levels of keto acids associated with group with poor SPPB scores (Additional file 1: Figure S2 B)

At discharge, pathway analysis documented alterations in the tricarboxylic acid (TCA) cycle, suggesting disrupted cellular energy production with potential adverse effects on physical recovery in patients with poor SPPB scores. Alanine, aspartate and glutamate metabolism and glycerophospholipid metabolism are also key pathways dysregulated at discharge (Fig. 2C).

Based on the ChemRICH analysis, carnitines emerged as the most elevated, while saturated fatty acids were the most decreased class of metabolites in patients with poor physical performance at the discharge time point (Additional file 1: Figure S2 C).

Although several pathways, including “Valine, leucine, and isoleucine degradation” and “Primary bile acid biosynthesis,” showed as dysregulated at earlier time points (ICU admission and day 5), their biological impact scores were limited, indicating subtle metabolic shifts. In contrast, pathway impact scores notably increased at discharge (Fig. 2).

Meta-Analysis: We were interested in determining if patient demographics (age, race, and sex) were associated with metabolomic changes. Overall, there was no association with age. On day 1, the only differential metabolite that was slightly downregulated in Black patients was 3-indoxyl sulfate.

Females exhibited higher levels of the bile acid taurocholate at both day 1 and day 5 compared to males. 1-palmitoyl-2-palmitoleoyl-GPC, was also differentially elevated in White patients than in Black patients on day 5.

At discharge, the metabolite profile showed no gender-based differential abundance. However, four metabolites were elevated in Black patients and three in White patients. Black patients showed higher levels of bile acids (taurochenodeoxycholate, hyocholate), threonate, and the peptide HWESASXX, which has been linked to worse outcomes in COPD [55]. In contrast to Black patients, White patients showed elevated levels of carnitine conjugates of medium and long chain fatty acids [(myristoleoylcarnitine (C14:1), palmitoleoylcarnitine (C16:1), 5-dodecenoylcarnitine (C12:1))], metabolites related to β-oxidation and mitochondrial function.

We were also interested in the potential influence of co-existing illness on the metabolic profile of the ARF patients that may be related to poor or good physical function. No significant associations were observed between patient physical function and comorbidities, APACHE III scores, or other clinical measures such as mean arterial pressure (Additional file 1: Figure S3).

Patient Classification Modeling: Given that the most differential serum metabolomic differences were observed at patient discharge compared to day 1 and day 5, we focused on this time point using the data of 30 participants with poor SPPB scores and 34 with good SPPB scores (Additional file 1: Figure S4A – S4C). We developed classification models to assess whether metabolomic profiles could anticipate the six-month SPPB scores. To identify the most relevant metabolites, we applied PLS-DA, Spearman correlation matrix, random forest feature selection, and evaluated using Bayesian leave-one-out crossvalidation (Additional file 1: Figure S4). Seven predictive metabolite biomarkers were selected for further analysis: cystine, glycerol 3-phosphate, hyocholate, 2-hydroxy-3-methylvalerate, taurocholenate sulfate, 1-oleoyl-2-docosahexaenoyl-GPC (18:1/22:6), and ximenoylcarnitine. These were subsequently used in a standard logistic regression model to assess their predictive performance. The standard logistic regression model achieved an accuracy of 0.86, along with a strong area under the curve (AUC) of 0.94. Following 5-fold cross-validation, the model continued to perform well, with an accuracy of 0.75 and an AUC of 0.86 (Additional file 1: Figure S5). These results suggest good discriminative ability without evidence of overfitting and support its potential to generalize to independent validation cohorts.

**Figure 3:**
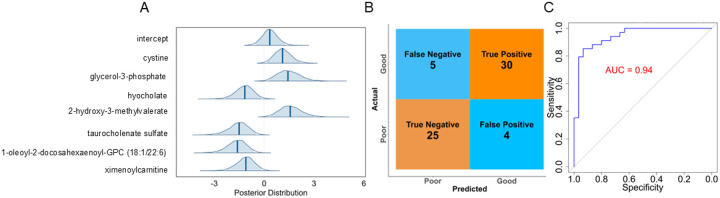
Bayesian Logistic Regression Model Classifying ARF Survivors Based on Selected Serum Metabolites at Discharge. Figure 3. Bayesian logistic regression model for classification of SPPB outcome (“Good” vs. “Poor”) at discharge using a seven-metabolite panel. Normal (0, 2.5) priors were placed on the intercept and all coefficients to balance regularization with data-driven inference. (A) Posterior distributions of each metabolite’s log-odds coefficient (vertical lines = medians; shaded regions = 95% credible intervals). Positive values indicate increased odds of a “Good” SPPB score; negative values indicate decreased odds. (B) Confusion matrix highlighting the Bayesian model’s prediction accuracy (C) ROC curve demonstrating robust predictive performance (AUC = 0.94).

To compare with standard logistic regression, we assessed the predictive capacity of the seven metabolites using Bayesian logistic regression framework. The intercept’s posterior distribution of the posterior plot, centered near zero, suggests baseline equal odds of ‘Poor’ versus ‘Good’ physical condition when metabolites are average (Fig. 3A). A slightly positive posterior indicates a mild bias toward predicting ‘Good’ SPPB scores. The posterior distributions for cystine, glycerol 3-phosphate, and 2-hydroxy-3-methylvalerate lie predominantly above zero, indicating that higher levels of these metabolites credibly increase the odds of a ‘Good’ SPPB score. In contrast, the posteriors for hyocholate, taurocholenate sulfate, 1-oleoyl-2-docosahexaenoyl-GPC, and ximenoylcarnitine lie below zero, suggesting that elevated concentrations are associated with poorer physical function. All metabolites present moderate posterior distributions with 95% credible intervals clear of zero, underscoring consistent associations in predicting physical function (Fig. 3A). The posterior predictive plot (Additional file 1: Figure S6 A) shows a close correspondence between simulated and observed outcomes, with the predictive intervals tightly enveloping the empirical data. This concordance indicates that the Bayesian model faithfully captures the underlying data structure and produces reliable predictions.

**Figure 4:**
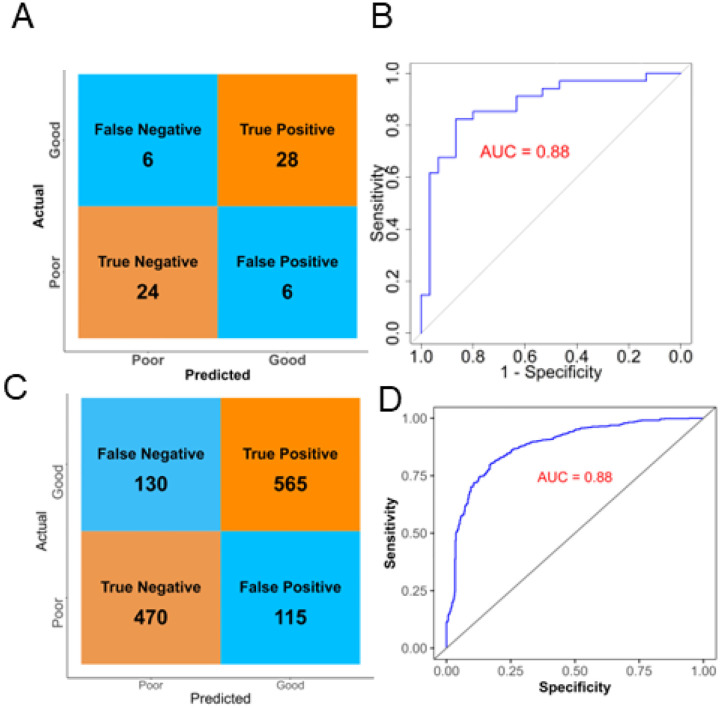
Cross Validation of Bayesian Model Classifying ARF Survivors. Performance evaluation of the Bayesian logistic regression model predicting SPPB outcomes (“Good” vs. “Poor”) based on serum metabolites measured at hospital discharge. (A) Confusion matrix and (B) Receiver Operating Characteristic (ROC) curve illustrating the performance from 5-fold cross-validation (AUC = 0.88). (C) Confusion matrix and (D) ROC curve derived from 100 model evaluations (repeated [20×5-fold] cross-validation (AUC = 0.88).

The confusion matrix and ROC curve of Bayesian model (Fig. 3B and 3C) indicate strong model-fit for classifying patients into ‘Good’ and ‘Poor’ SPPB groups [56]. The trace plot indicates convergence to the target distribution, suggesting well-sampled posterior distributions (Additional file 1: Figure S6 B). Low autocorrelation in MCMC chains implies efficient Bayesian sampling, with reliable posterior estimates (Additional file 1: Figure S6 C) [57]. Patient classification under the Bayesian framework was further assessed using 5-fold and repeated k-fold cross-validation (100 model evaluations), achieving a robust AUC of 0.88 (Fig. 4). Collectively, these results underscore strong predictive associations between serum metabolites measured at hospital discharge and subsequent patient physical function. The consistently high AUC values and accuracy across multiple validation approaches indicate that the selected metabolite panel provides stable and effective predictive utility for future patient physical performance outcomes.

**Figure 5:**
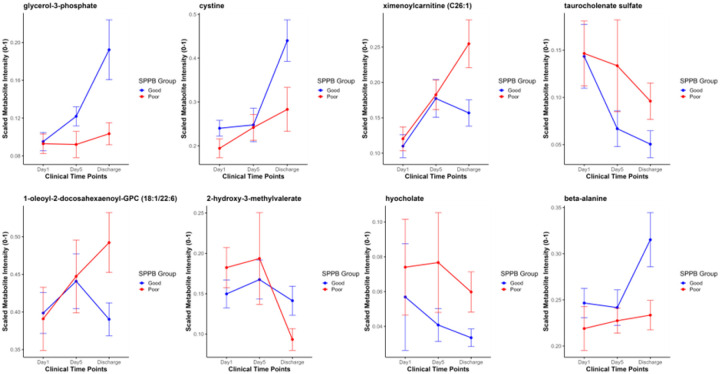
Line Plot of SPPB Predictive Metabolites and Beta-Alanine. Longitudinal changes in selected serum metabolites across three clinical time points, day 1 (ICU admission), day 5 post-admission, and hospital discharge, stratified by six-month physical function (SPPB) groups. Blue lines and points denote patients with “Good” SPPB scores; red lines and points denote those with “Poor” SPPB scores. Data points are group means and error bars represent ± standard error of mean (SEM). Each panel shows one metabolite as labeled in the title, 7 metabolites predictive of six-month SPPB outcomes, and beta-alanine which has an established role in muscle metabolism.

The line plot illustrates the trends of the seven predictive metabolites biomarkers and β-alanine at key time points during hospitalization, day 1 of ICU admission, day 5, and at discharge (Fig. 5).

## Discussion

Based on our prior work in ARF patients, we hypothesized that the most pronounced metabolomic differences between patients with good and poor physical function would occur at ICU admission, reflecting the acute severity of illness. However, our findings revealed a surprising pattern: despite clinical improvement by the time of discharge, the number of differential metabolites was threefold higher at discharge compared to ICU admission or mid-hospitalization (Additional file 1: Figure S7). The only metabolite that consistently differed at all three time points between patients with poor and good physical function was the bile acid taurochenodeoxycholate, showing higher mean and median values in those with poor SPPB test results. Additionally, bile acids were generally elevated across all time points in patients with poor physical performance, suggesting a consistent dysregulation of bile acid metabolism in this group. These findings are further supported by hierarchical clustering of multivariate metabolite profiles, which revealed some noticeable patterns between good and poor SPPB groups across time points, especially in differential metabolites related to bile acid and phosphatidylethanolamines metabolism and bioenergetics (Additional file 1: Figure S1).

ChemRICH and pathway analysis (using MetaboAnalyst) identified similar, though slightly varied dysregulated metabolomic alterations at each time point. These findings suggest that while the overall metabolic disruptions are consistent, the specific pathways and metabolites involved may shift as the patient progresses through the phases of hospital care and recovery. Our previous research demonstrated a correlation between acute mortality in ARF patients and elevated carnitine levels alongside mitochondrial-related bioenergetic dysfunction [30]. Notably, ChemRICH analysis of metabolites at discharge revealed that carnitines constituted the most enriched biochemical set in patients with poor SPPB scores, supporting the association between carnitine upregulation and worse outcomes in critically ill patients (Additional file 1: Figure S2 C) [30]. Patients with poor physical function showed reduced lipid and fatty acid levels, and elevated bile acids (Fig. 1C; Additional file 1: Figure S2 A, S2 C), consistent with prior studies linking low lipid levels in critical illness to worse outcomes [58,59]. These metabolomic differences suggest a shift in bile acid homeostasis, potentially reflecting altered hepatic metabolism, although serum albumin and bilirubin levels did not differ significantly between groups (Table 1) [60–63].

At the discharge time point, disruption in glycerophospholipid metabolism in the poor SPPB group may impair membrane lipid turnover, which is critical for maintaining cellular integrity and membrane fluidity. Similar reductions in glycerophospholipid metabolism have been previously observed to correlate with poor outcomes in patients with COPD (Fig. 2C and Additional file 1: Figure S1) [64].

Nutritional status variations also could affect the metabolic disparities between patients with high and low SPPB scores [65]. The lower fatty acid and lipids levels observed with poor SPPB scores suggest that the increased bile acid production may be a compensatory metabolic response, potentially important for enhancing lipid digestion and absorption. On day 1, 3-indoxyl sulfate, a tryptophan-derived metabolite was downregulated in Black ARF survivors and has been implicated in microbial–host interactions, gut homeostasis, and uremic toxicity [66,67]. Moreover, a diet modifiable metabolites, 1-palmitoyl-2-palmitoleoyl-GPC, was differentially elevated in White patients than in Black patients on day 5 [68]. Given that only few ARF patients in the cohort received nutritional therapy, and considering the known impact of nutritional status on both short- and long-term outcomes in critical illness, metabolomic profiling may offer valuable insights into abnormal metabolic signatures. These insights could inform personalized nutritional strategies to improve long-term functional outcomes in ARF patients [69,70].

It is also noteworthy that the APACHE III score showed no significant association with poor physical performance. While this clinical metric provides an overall assessment of patient condition, it may not capture the nuanced biological variability that influences physical function in critically ill patients. Likewise, other important variables such as physical therapy, home oxygen use, and dialysis have no significant association with poor or good physical function. The lack of significant association in aforementioned parameters with the physical function status may be due to modest sample size used in this preliminary study. On the contrary, patients with longer ICU stays were significantly more likely to have poor physical function outcomes (Table 1).

We observed decreased levels of β-alanine at discharge in patients with poor SPPB scores (Fig. 1C and 2C). β-alanine is a central metabolite serving as a precursor in various key metabolic processes, including the synthesis of carnosine, a dipeptide of β -alanine and histidine, which supports muscle metabolism [71–75]. β-alanine intake could thus potentially benefit ARF patients prone to poor physical performance and thus seem to warrant additional research into its clinical applications [71,72,74–76].

The strong performance of both logistic regression and Bayesian logistic regression models demonstrates that metabolomic profiling at the time of ICU discharge can effectively predict patients’ subsequent physical performance (Additional file 2: Table S1). While frequentist logistic regression shares a similar foundational framework with its Bayesian counterpart, the Bayesian approach demonstrated superior performance during cross-validation, achieving an AUC of 0.88 compared to 0.86 with standard logistic regression. Moreover, the Bayesian model yielded fewer false positives and false negatives in sample classification at cross validation (Fig. 4 and Additional file 1: Figure S5). To our knowledge, this represents the first statistically grounded machine learning model that leverages metabolomic data to classify and predict future physical function outcomes in survivors of ARF.

Some metabolites with predictive utility (2-hydroxy-3-methylvalerate, hyocholate, cystine, and β-alanine) showed temporal trends and partial separation between good and poor SPPB groups at day 1 or day 5, but none were considered differential (nominal p > 0.05) at these time points (Fig. 5 and Additional file 1: Figure S7). Independent validation of the predictive performance of the Bayesian logistic regression model is necessary to further strengthen the case for the application of these metabolites to predict PICS of discharged ARF patients.

The biomarkers identified as predictive of physical function in this study are FDR-independent, as they were selected based on multivariate modeling rather than univariate significance [77,78]. A limitation of this study is that the metabolites identified differential (p < 0.05) did not retain significance after correction for multiple testing (adjusted p > 0.05) (see Additional File 3). This may be partly attributable to the modest sample size. As part of our next step, determining absolute metabolite concentrations through calibration to internal and external standards in a targeted metabolomics approach will help minimize variability in metabolite quantification and with increased sample size and statistical power, thereby strengthening the validation of these findings [79].

This pilot study was designed to maximize the use of available resources and to guide the identification of metabolites for focus in future, more targeted investigations. We were also limited by the number of patients that had the final 6-month SPPB scores. Future prospective studies may need to utilize different tools for determining physical function, such as activity tracker watches that do not require direct patient interaction after patient discharge. While the predictive modeling and cross-validation results are promising, the absence of an independent validation cohort limits our ability to assess the model’s generalizability and stability.

Increased hospital length of stay among ARF survivors enrolled in this study was significantly associated with poor SPPB scores, indicating worse physical function. This variability in discharge timing may have introduced some heterogeneity into the metabolomic findings, particularly if longer hospitalization reflects differing stages of recovery or persistent metabolic impairment. Importantly, the relatively longer LOS in survivors with poor physical performance aligns with our postulate of a continuum of bioenergetic dysregulation. Non-survivors in our prior studies exhibited severe mitochondrial dysfunction and global metabolic collapse, the survivors with diminished physical function in this cohort may represent an intermediate phenotype, marked by sub-lethal, bioenergetic deficits that impair recovery [29–34].

All patients in the study cohort received standard of care which included various medications, that may have some influence on the metabolomic profile. We did not stratify outcomes based on the underlying etiology of ARF (infectious vs. non-infectious), which may have contributed to variability in the observed metabolomic and functional outcomes. This study was retrospective; future prospective studies are necessary to confirm the clinical utility of the predictive model. Targeted metabolomics, particularly for NAD^+^, glycolysis, and mitochondrial redox pathways, may improve sensitivity and specificity in future analyses. Longitudinal monitoring of post-discharge metabolomic profiles would help determine whether these pathways remain significantly altered in patients with poor outcomes and could also assess the impact of interventions such as diet and physical therapy on metabolic recovery. In the next phase of our research, we will conduct prospective validation in independent ARF cohorts across multiple centers with increased sample size. These studies will also incorporate socioeconomic factors and data on access to, and engagement in, physical therapy, thereby providing a more comprehensive understanding of the determinants of physical recovery after ICU discharge.

## Conclusions

Metabolomic profiling of bioenergetic-related metabolites at ICU discharge can distinguish ARF survivors with good versus poor physical performance. We propose that serum metabolomic analysis at discharge offers actionable clinical insight into a patient’s metabolic status and recovery trajectory. Quantitative targeted measurement of key bioenergetic and bile acid metabolites may enable risk stratification, inform nutritional or rehabilitative interventions, and personalize post-ICU care. While future prospective studies are required to validate the accuracy and clinical utility of this model, the predictive metabolomic panel identified in this study holds strong potential as a prognostic tool to identify patients at high risk for poor physical recovery and to guide targeted interventions aimed at improving long-term outcomes.

## Supplementary Material

This is a list of supplementary files associated with this preprint. Click to download.

- Additionalfile1.pptx
- Additinalfile2.xlsx
- Additinalfile3.xlsx

## Figures and Tables

**Table 1:** Patients Demographics

Variable	Good	Poor	P-value
n-value	35	35	
SPPB score	10 [8.5–10]	3 [0–5]	<0.001^a^
APACHE III score	70 [57.5–82.5]	69 [60.5–79]	0.764^a^
MAP (mmhg)	65 [56–93]	68 [57–100]	0.477^a^
Pulse (bpm)	103 [85–123]	106 [85.5–114]	0.577^a^
Respiratory rate (breaths/min)	24 [20.5–30.5]	22 [19.5–28.5]	0.303^a^
PCO_2_ (mmhg)	44 [34–52.25]	37.6 [32.5–43.7]	0.127^a^
PAO_2_ (mmhg)	95 [77–121]	91.6 [75.05–112.5]	0.703^a^
FIO_2_ (mmhg)	0.5 [0.4–0.6]	0.6 [0.4–0.7]	0.386^a^
BUN (mg/dl)	19 [15–29]	18 [11.5–28]	0.577^a^
Serum albumin (g/dl)	3 [2.6–3.3]	2.7 [2.3–3.2]	0.127^a^
Serum bilirubin (mg/dl)	0.8 [0.6–1.05]	0.9 [0.55–1.15]	0.991^a^
Blood glucose (mg/dl)	137 [100–204]	132 [116.5–164.5]	0.707^a^
BMI (kg/m^2^)	30.27 [26.6–36.33]	29.67 [23.07–34.35]	0.280^a^
Age (years)	62 [54–69.5]	59 [54–71.5]	0.986^a^
LOS (days)	9 [7–11.5]	12 [8.5–17]	0.019^a^
Sex			1^b^
Female	18 (51.4%)	18 (51.4%)	
Male	17 (48.6%)	17 (48.6%)	
Race			1^b^
Black	11 (31.4%)	11 (31.4%)	
White	24 (68.6%)	24 (68.6%)	
Nutrition			1^b^
No	30 (85.7%)	29 (82.9%)	
Yes	5 (14.3%)	6 (17.1%)	
Home oxygen			1^b^
No	25 (71.4%)	25 (71.4%)	
Yes	10 (28.6%)	10 (28.6%)	
Dialysis			1^b^
No	31 (88.6%)	33 (94.3%)	
Yes	4 (11.4%)	2 (5.7%)	
Physical therapy			1^b^
No	15 (42.9%)	15 (42.9%	
Yes	20 (57.1%)	20 (57.1%)	

## Data Availability

The dataset supporting the conclusions of this article is included within the article and its additional files. The code used to process and analyze the metabolomics data is publicly available at https://github.com/RNABioUSA/arfqol-metabolomics.

## Abbreviations

SPPB: short physical performance battery
ARF: acute respiratory failure
UHPLC-MS: ultra-high performance liquid chromatography–mass spectrometry
ICU: intensive care unit
COPD: chronic obstructive pulmonary disease
ARDS: acute respiratory distress syndrome
PICS: post-intensive care syndrome
PLS-DA: partial least squares discriminant analysis
APACHE: Acute Physiology and Chronic Health Evaluation
TCA: tricarboxylic acid cycle
ROC: receiver operating characteristic curve
AUC: area under the curve
